# Explaining multivariate molecular diagnostic tests via Shapley values

**DOI:** 10.1186/s12911-021-01569-9

**Published:** 2021-07-08

**Authors:** Joanna Roder, Laura Maguire, Robert Georgantas, Heinrich Roder

**Affiliations:** Biodesix, Inc., 2970 Wilderness Place, Ste100, Boulder, CO 80301 USA

**Keywords:** Explainability, Shapley values, Molecular diagnostic test, Machine learning, Artificial intelligence, Interpretability

## Abstract

**Background:**

Machine learning (ML) can be an effective tool to extract information from attribute-rich molecular datasets for the generation of molecular diagnostic tests. However, the way in which the resulting scores or classifications are produced from the input data may not be transparent. Algorithmic explainability or interpretability has become a focus of ML research. Shapley values, first introduced in game theory, can provide explanations of the result generated from a specific set of input data by a complex ML algorithm.

**Methods:**

For a multivariate molecular diagnostic test in clinical use (the VeriStrat® test), we calculate and discuss the interpretation of exact Shapley values. We also employ some standard approximation techniques for Shapley value computation (local interpretable model-agnostic explanation (*LIME*) and Shapley Additive Explanations (*SHAP*) based methods) and compare the results with exact Shapley values.

**Results:**

Exact Shapley values calculated for data collected from a cohort of 256 patients showed that the relative importance of attributes for test classification varied by sample. While all eight features used in the VeriStrat® test contributed equally to classification for some samples, other samples showed more complex patterns of attribute importance for classification generation. Exact Shapley values and Shapley-based interaction metrics were able to provide interpretable classification explanations at the sample or patient level, while patient subgroups could be defined by comparing Shapley value profiles between patients. *LIME* and *SHAP* approximation approaches, even those seeking to include correlations between attributes, produced results that were quantitatively and, in some cases qualitatively, different from the exact Shapley values.

**Conclusions:**

Shapley values can be used to determine the relative importance of input attributes to the result generated by a multivariate molecular diagnostic test for an individual sample or patient. Patient subgroups defined by Shapley value profiles may motivate translational research. However, correlations inherent in molecular data and the typically small ML training sets available for molecular diagnostic test development may cause some approximation methods to produce approximate Shapley values that differ both qualitatively and quantitatively from exact Shapley values. Hence, caution is advised when using approximate methods to evaluate Shapley explanations of the results of molecular diagnostic tests.

**Supplementary Information:**

The online version contains supplementary material available at 10.1186/s12911-021-01569-9.

## Background

Multiplexed measurement methods from the fields of proteomics, genomics, transcriptomics, and metabolomics can generate vast amounts of patient data, which can be used to predict patient outcome with the application of machine learning (ML) techniques. Molecular diagnostic tests produced from large numbers of attributes via ML can be effective predictors of outcome, making use of the information in these highly multivariate data inputs to improve performance and robustness. However, neither the way in which the tests produce a result for a given patient, nor the biological rationale underlying the tests may be transparent. While translational studies can be used to elucidate the biological mechanisms behind such tests, providing a meaningful and accurate picture of how a test classification is arrived at, given the input data for a specific patient, requires a different approach.

Explainability of ML models and artificial intelligence (AI) has recently become a major focus of attention in other areas in which these methods are employed [1]. Concerns about biases in ML implementations, including those containing the attributes gender or race [2, 3], and the recognition of the right of individuals to understand how their personal data is being used, have highlighted the need for interpretable explanations and quantification of how attributes are used by complex ML algorithms [4].

Explainability research has moved away from trying to provide a global description of relative attribute importance accurate for all possible sets of input data, which is unlikely to be successful given the complexity and nonlinearities inherent in most ML approaches, to a local approach of assessing relative attribute importance for the prediction generated for a specific input data instance. In the case of molecular tests, this latter approach corresponds to providing a description of the relative importance of used attributes generating a result for an individual patient.

One proposal has been to construct a simpler, more interpretable model of a complex ML algorithm which reproduces the results of the full ML algorithm in the vicinity of the individual patient data for which we seek to explain the test result. The local interpretable model-agnostic explanation (*LIME*) approach seeks to generate a model of a ML algorithm which can be easily interpreted and which can reproduce the results of the ML algorithm in the locality of the input data without requiring information about the ML algorithm itself [5]. Only oracle access to the ML algorithm is required—i.e., only results of the ML algorithm for various inputs are necessary, not any details of how they are arrived at from the input attributes.

An alternative approach to explainability makes use of game theory concepts developed for determining the equitable distribution of winnings between players working in teams [6, 7]. Several years ago, parallels were observed between deciding on a fair distribution of winnings between team members in multiplayer games and assessing the relative importance to the result of a ML algorithm of multiple input attributes [8–10]. It was proposed that Shapley values (SVs), which provide an equitable scheme for dividing game winnings within a team of multiple players, could provide the framework for assigning relative importance of multiple attributes to the result of a ML algorithm. SVs assess the contribution of a player to the team’s result, or an attribute to the algorithm output, by examining the results for all possible coalitions of players within the team, or subsets of possible attributes. SVs satisfy several axioms (discussed in Methods) which guarantee that these explanations possess some desirable properties [6, 11, 12]. Unfortunately, exact calculation of SVs is generally unfeasible in terms of computation time and requires detailed knowledge of the ML algorithm, which may not be available. Recent research has focused on methods for approximating SVs which circumvent these issues. These strategies include Shapley Additive Explanations (*SHAP*) [13, 14], Kernel *SHAP* [13], and methods where calculation of the exponentially large number of coalitions that must be evaluated for calculation of an exact SV is replaced by sampling only a subset of these coalitions [9, 10, 13, 15].

While these explainability approaches have proved useful in certain AI applications, notably in natural language processing, image-related, and other traditional Big Data tasks [1], applying them to molecular diagnostic ML algorithms presents some potential difficulties. Training sets for molecular diagnostics ML-algorithms are usually very restricted in number of observations (patients or samples). In addition, the training sets are sometimes chosen to be enriched in certain patient subsets (e.g., responders vs progressors on a particular therapy), rather than being representative of the population on which the test will be applied. Hence, methods employing distributional approaches can be problematic. Furthermore, molecular and clinical data are often highly correlated. While these correlations can be used to advantage, for example in set enrichment methods, they present additional challenges to some SV approximation methods that do not maintain inter-feature correlations [12]. Finally, most ML algorithms produce results that are either binary, have a limited number of categories, or are bounded scores. Hence, the winnings of the equivalent game are bounded or take a limited number of discrete values, rather than being continuous and unbounded. As a result, the value range on which Shapley values are measured is limited leading to interpretational difficulties [12].

Our goal in this manuscript is to demonstrate how exact SVs can be used to explain the results of one example of a clinically used molecular diagnostic test and how they can be interpreted. We also apply the *LIME* and *SHAP*-based approaches, which are available in standard ML software packages (IBM’s AI Explainability 360, Anaconda Python package (https://anaconda.org/powerai/aix360), Matlab’s Deep Learning Toolkit) and increasingly used in applications, to the same test to explore the differences between the results of these approximate methods and exact SVs. Further, we apply three methods for assessing influence of pairs of attributes, rather than individual attributes, within the SV framework: Harsanyi Dividends (*HDs*) [7], Shapley Interaction Indices (*SIIs*) [14], and Shapley-Taylor Interaction Indices (*STIIs*) [16]. We discuss their interpretation and propose that alternative quantities may be required to provide information on the contribution to test results of one attribute in addition to another specific attribute that are useful in the context of binary molecular diagnostic tests.

## Methods

### The VeriStrat test

Our studies use the VeriStrat® (VS) test as an example of a molecular diagnostic test [17]. The test has been performed on more than 40,000 patient samples in a routine clinical setting and has been prospectively validated for its prognostic value and ability to predict differential outcome between chemotherapy and targeted therapy for patients with non-small cell lung cancer (NSCLC) [18]. The test has also been shown to have prognostic power for prediction of outcomes for patients with several other cancer types [19, 20] and for patients with NSCLC treated with other therapies [21].

The VS test is a mass spectrometry-based proteomic test run on a blood-based sample. Full details of test development and validation have been provided elsewhere [17, 22]. Three technical replicate spectra are generated from each sample. Each replicate spectrum is processed to render the data reproducible and comparable between samples, and feature values from eight mass/charge spectral regions, centered at 5843, 11,445, 11,529, 11,759, 11,903, 12,452, and 12,579, respectively, evaluated. These feature values are combined using a 7-nearest neighbor (7NN) classifier based on a reference set of feature values from 26 samples, 13 defined as Poor (derived from patients with NSCLC experiencing rapid progression on gefitinib therapy) and 13 defined as Good (derived from patients with NSCLC experiencing long term stable disease on gefitinib therapy), to produce a Poor or Good classification for each technical replicate. When all three replicates produce concordant classifications, a result of VS Good (likely to experience better outcomes) or VS Poor (likely to experience worse outcomes) is reported. Non-concordant replicate classifications result in a VS Indeterminate result. Here, we focus on the result obtained from applying the VS algorithm to one instance of data from a single technical replicate mass spectrum (an “instance” that will be classified as either Good or Poor). The VS ML algorithm is simple enough to permit calculation of exact SVs and exact interaction indices for a large cohort of patients and to allow comparisons with *SHAP* values and the results of the *LIME* approach.

The VS features are known to be strongly correlated (off-diagonal correlation matrix elements vary from 0.310 to 0.996 for the 256-instance cohort considered in this study), with multiple features corresponding to isoforms of C-terminal truncation of serum amyloid A (SAA) [23, 24]. The 11,529 feature is also understood to contain doubly-charged C-reactive protein. Increased levels of inflammatory proteins have been observed in patients with VS Poor classification [25].

### Patients and samples

The cohort for SV evaluation constituted 256 patients from the phase 3 prospective clinical trial validating the ability of the VS test to predict differential outcomes between single agent chemotherapy and targeted therapy in second-line NSCLC [18]. All patients provided written informed consent and the study was approved by institutional review boards and independent ethics committees at the 14 participating sites. The sample aliquots had been processed and classified during the trial concordance analysis. Trial inclusion and exclusion criteria and patient baseline characteristics have been previously published [18]. While all patients included in the cohort received a classification of VS Good (N = 179) or VS Poor (N = 77) from initial trial aliquot, the aliquots used for this study yielded 186 VS Good classifications, 64 VS Poor classifications and 6 VS Indeterminate results.

In this study we were primarily interested in the classifications produced for the first technical replicate (instance) generated from each aliquot. Of the 256 clinical trial patients with concordance study aliquots, 67 instances were classified as Poor and the remaining 189 as Good.

### Exact Shapley values

SVs assess the contribution of a player to the team’s result, or the contributions of an attribute to the algorithm output, by examining the results/algorithm predictions for all possible coalitions of players within the team, or all possible subsets of attributes [6]. Formally, let us assume that we have a predictor *f*(*M*) which depends on a set of attributes, *M*. Further, we assume that we can define the prediction for any subset of attributes, *S*, contained within a set of all available attributes *M*. The SV for an attribute *j* contained in *M* is

1 $$\begin{array}{*{20}c} {\psi _{j} \left( f \right) = ~\mathop \sum \limits_{{S~ \subseteq M\backslash \left\{ j \right\}}} \frac{{\left| S \right|!\left( {\left| M \right| - \left| S \right| - 1} \right)!}}{{\left| M \right|!}}~\left( {f\left( {S \cup \left\{ j \right\}} \right) - f\left( S \right)} \right)} \\ \end{array}$$

It has been demonstrated that SVs satisfy several axioms which have intuitive interpretations in the molecular diagnostic test setting:

- *Efficiency or local accuracy* The sum of the SVs over all attributes, including the null set, is the algorithm prediction, i.e., $\sum\nolimits_{{j = 0}}^{{\left| M \right|}} {\psi _{j} } \left( f \right) = ~f\left( M \right).$
- *Symmetry* Two attributes that contribute equally to all possible coalitions have equal SVs. i.e., if $f\left( {S \cup \left\{ j \right\}} \right) = ~f\left( {S \cup \left\{ i \right\}} \right)$ for every subset *S* not including attributes *i* or *j*, $\psi _{j} \left( f \right) = \psi _{i} \left( f \right)$.
- *Dummy player or missingness* An attribute that contributes nothing to any coalition has zero SV. i.e., if $f\left( {S \cup \left\{ j \right\}} \right) = ~f\left( S \right)$ for every subset *S* not including *j*, $\psi _{j} \left( f \right) = 0.$

In the case of our investigation of the SVs of the VS test, the predictor *f* is a 7NN classifier using a 26-member reference set. Possible output predictions are *f* = −1 (corresponding to a Poor classification) or *f* = 1 (a Good classification). Exact SVs were calculated using 7NN classifiers with all 2^8^ (= 256) possible subsets of the eight features from Eq. 1. The value of *f* when no features are used (the null set) is defined as uninformative, i.e., $f\left( {\left\{ \emptyset \right\}} \right) = 0.$

### Exact Shapley interaction indices

In molecular diagnostics studies, the question sometimes arises what information feature *i* adds to another feature *j*. Alternatively, we may be interested in predictive information contained in the interaction of two features, for example an outcome that can be predicted by a low value of feature *i* only when feature *j* has a high value. We investigated three expressions proposed to characterize the importance of pairs of features, or interactions, for classification: Shapley interaction indices [14], Shapley-Taylor interaction indices [16], and two-feature Harsanyi dividends [7].

#### Shapley interaction indices (SIIs)

The *SII* for features $i,j~\left( {i \ne j} \right)$ [14] is defined as

2 $$SII_{{ij}} \left( f \right) = \sum\limits_{{S~ \subseteq M\backslash \left\{ {i,j} \right\}}} {\frac{{\left| S \right|!\left( {\left| M \right| - \left| S \right| - 2} \right)!}}{{2\left( {\left| M \right| - 1} \right)!}}~\left[ {f\left( {S \cup \left\{ {i,j} \right\}} \right) - f\left( {S \cup \left\{ i \right\}} \right) - f\left( {S \cup \left\{ j \right\}} \right) + f\left( S \right)~} \right]}$$

A main effects term, $SII_{{ii}} \left( f \right)$ can be defined as the difference between $\psi _{i}$ and the sum over all interaction terms involving feature *i*:

3 $$\begin{array}{*{20}c} {SII_{{ii}} \left( f \right) = ~\psi _{i} - ~\mathop \sum \limits_{{j \ne i}} SII_{{ij}} \left( f \right).~} \\ \end{array}$$

#### Shapley-Taylor interaction indices (STIIs)

The *STII* for the subset of two features $i,j~\left( {i \ne j} \right)$ [16] is defined as

4 $$STII_{{ij}} \left( f \right) = ~\mathop \sum \limits_{{S~ \subseteq M\backslash \left\{ {i,j} \right\}}} \frac{{2\left| S \right|!\left( {\left| M \right| - \left| S \right| - 1} \right)!}}{{\left| M \right|!}}~\left[ {f\left( {S \cup \left\{ {i,j} \right\}} \right) - f\left( {S \cup \left\{ i \right\}} \right) - f\left( {S \cup \left\{ j \right\}} \right) + f\left( S \right)~} \right].~$$

A main effects term for $i = j$ [16] can also be defined as

5 $$STII_{{ii}} \left( f \right) = ~\left[ {f\left( {\left\{ i \right\}} \right) - f\left( \left\{ \emptyset \right\} \right)~} \right].$$

Note that [16] defines the *STIIs* and *SIIs* for a subset of two features rather than for a pair of indices. Hence, [16] defines *SIIs* as twice the quantity in Eq. 2. In this paper, we maintain the original definitions of *SIIs* from [14] and *STIIs* from [16].

#### Harsanyi dividends (HDs)

The *HD* for two features $i,j~\left( {i \ne j} \right)$ [7] is defined via the expression

6 $$\begin{array}{*{20}c} {HD_{{ij}} \left( f \right) = f\left( {\left\{ {i,j} \right\}} \right) - f\left( {\left\{ i \right\}} \right) - f\left( {\left\{ j \right\}} \right) - 2f\left( {\left\{ \emptyset \right\}} \right).} \\ \end{array}$$

#### Shapley partial sums (SPSs)

In addition to the three expressions above, we defined a fourth quantity to capture the impact of features $i,j~\left( {i \ne j} \right)$ on classification, the Shapley partial sum, *SPS*. *SIIs*, *STIIs*, and *HDs* are all symmetric in *i* and *j* by construction. It is generally unlikely that the impact on a classifier of adding feature *i* to feature subsets including feature *j* will be identical to that of adding feature *j* to feature subsets including feature *i*, and it may be of interest to assess these two quantities.

Note that $\psi _{j} \left( f \right)~$ can be split into two separate sums, one containing only feature subsets including feature *i* and one containing only feature subsets not including feature *i*, Eq. 7.

7 $$\begin{aligned} \psi _{j} \left( f \right) & = ~\mathop \sum \limits_{{S \subseteq M\backslash \left\{ {i,j} \right\}}} \frac{{\left| {S + 1} \right|!\left( {\left| M \right| - \left| {S + 1} \right| - 1} \right)!}}{{\left| M \right|!}}~\left( {f\left( {S \cup \left\{ {i,j} \right\}} \right) - f\left( {S \cup \left\{ i \right\}} \right)} \right) \\ & \quad + \,\mathop \sum \limits_{{S~ \subseteq M\backslash \left\{ {i,j} \right\}}} \frac{{\left| S \right|!\left( {\left| M \right| - \left| S \right| - 1} \right)!}}{{\left| M \right|!}}~\left( {f\left( {S \cup \left\{ j \right\}} \right) - f\left( S \right)} \right) \\ \end{aligned}$$

The first term in Eq. 7 captures the contribution of feature *j* to coalitions including feature *i*, i.e., the contribution to the classification of feature *j* when used together with feature *i*. Hence, we defined the $SPS_{{ij}} ~\left( f \right)$ for $~i \ne j$ as

8 $$SPS_{{ij}} \left( f \right) = \sum\limits_{{S~ \subseteq M\backslash \left\{ {i,j} \right\}}} {\frac{{\left( {\left| M \right| - \left| S \right| - 2} \right)!\left( {\left| S \right| + 1} \right)!}}{{\left| M \right|!}}~\left[ {f\left( {S \cup \left\{ {i,j} \right\}} \right) - f\left( {S~ \cup ~\left\{ i \right\}} \right)~} \right]~.~}$$

Note that, unlike the three kinds of interaction evaluated above, *SPS*_*ij*_ is not symmetric in the indices *i* and *j*, i.e., $SPS_{{ij}} ~\left( f \right) \ne ~SPS_{{ji}} ~\left( f \right)$. This corresponds to the notion that if we have two correlated features that share some information useful for classification, but one of the features is superior in information content to the other, the benefit of adding the lower information content feature once we have already used the higher information content feature is smaller than the benefit of adding the higher information content feature once we have already used the lower information content feature.

### *SHAP* methods

*SHAP* approaches are based on replacing retraining of the classifier on feature subsets with conditional expectations of the original algorithm result [13]. Formally, if we consider the case where the input data is a vector, ***x***, of dimension $\left| M \right|,$ and our algorithm is $f\left( M \right) = g\left( \user2{x} \right)$, $SHAP$ replaces the prediction $f\left( {\left\{ S \right\}} \right)$ for a sample with input vector $\user2{x}^{\user2{*}}$, by $E{\text{[}}g\left( \user2{x} \right){\text{|}}\user2{~x}_{\user2{i}} = \user2{x}_{\user2{i}}^{\user2{*}} ,\user2{~}{\text{for}}~~i \subseteq \left\{ S \right\}]$ where $E\left[ . \right]$ is the expected value. While this approach seems intuitively very reasonable, it may not be clear what is meant by the expected result of the algorithm output conditional on the feature values being maintained within the subset *S* [26]. This is particularly problematic when the input data are continuous and the training set is small. While the training set necessarily defines the prediction algorithm, in cases of molecular diagnostics it may be unrepresentative of the population of samples to be classified and it is often small and of high dimensionality such that adequate estimation of the distribution over which to calculate the expectation can be difficult or impossible.

We tested three different *SHAP*-based approaches: kernel *SHAP* [13], which treats features as independent, and two versions of *SHAP* that allow incorporation of correlations between features, the multivariate Gaussian approximation and the Gaussian copula approximation [12]. These three approaches are described below in increasing order of complexity of implementation and capacity to approximate inter-feature correlations.

#### Kernel SHAP

Kernel *SHAP* values [13] were calculated following method 2.3.2 of [12]. The predictions *f*(*S*) in Eq. 1 were replaced by their expectation value in order to avoid retraining. In kernel *SHAP*, features are assumed to be independent (an inaccurate assumption in the case of the VS test and most -omics datasets, where correlations between features are common). For a given instance and feature subset *S*, the expectation value of *f*(*S*) is calculated using all 26 reference samples as follows: maintain instance feature values for features in *S*, replace instance feature values outside *S* by the feature values from one of the 26 reference samples, classify the results, repeat for the other 25 reference samples and average the 26 resulting classifications.

We note here that, as pointed out by Kumar et al. [26], replacing the features outside of *S* by feature values for these features from the training set, or by features drawn from a distribution defined by the training set, independently from the features in *S*, can lead to evaluations of the algorithm in regions of feature space which the algorithm never experienced during training and which may not even be spanned by real, physical samples. This is indeed the case for the VS algorithm where the correlations between features are strong.

#### Multivariate Gaussian feature distribution

The multivariate Gaussian *SHAP* approximation method incorporates feature dependence by assuming that the feature distribution is a multivariate Gaussian and estimating the parameters of the distribution from the training set. Given the Gaussian assumption, the distribution of features not in *S* conditional on the feature values of features in *S* can be explicitly calculated. The expectation value of a prediction *f*(*S*) is then calculated by drawing *n* samples from this conditional distribution, classifying the samples, and averaging the results. We followed method 3.1 in Aas et al. [12], using all 26 reference samples to estimate the parameters of the multivariate Gaussian distribution and drawing *N* = 1000 samples to estimate each expectation value.

#### Gaussian copula

The Gaussian copula *SHAP* approximation method builds on the multivariate Gaussian method to allow estimation of the conditional distribution for a non-normal feature distribution via transformation of variables with a probability integral transform (PIT) to a copula assumed to be Gaussian. We followed method 3.2 of Aas et al. [12], again using all 26 reference samples to estimate distribution parameters. Briefly, the multivariate feature distribution is mapped using the probability integral transform (PIT), maintaining pairwise correlations using the copula approach. Under the assumption that the copula is Gaussian, the distribution of features not in *S* conditional on the feature values of features in *S* can be evaluated. Samples can then be drawn from the resulting conditional distribution, mapped back via the inverse PIT and classified before averaging to give the conditional expectation. We drew *N* = 1000 samples to estimate each expectation value.

### LIME methods

*LIME* explanations were calculated following the method of Ribeiro et al. [5]. The *LIME* approach constructs a local interpretable model, i.e., a simplified model, that allows easy determination of feature importance and which reproduces the results of the more complex test at the feature space location of the sample, using data from the feature space vicinity of the sample for which the test result is to be explained. In this study, as we are working with binary classification, we selected logistic regression or a support vector machine (SVM) as the local interpretable model. In the case of the logistic regression modelling, the regression coefficients can be taken as representative of the relative importance of each feature for the classification of an instance. For a SVM, the coefficients defining the vector perpendicular to the SVM decision boundary provide a measure of relative feature importance for classification [27].

In implementing the *LIME* approach, the problem arose how to generate truly *local* models for instances lying within the bulk of Good or Poor classification regions of feature space. Instances with both classifications are required to train the local models, but in the bulk of one of the feature space classification regions, local variations about the point of interest in feature space only produce instances with the same classification. Hence, to be able to train a model, the definition of local must be relaxed to cover a substantial fraction of feature space; the resulting model is no longer a local one.

To make the coefficients obtained from the trained local models interpretable, the features used in the VS test were first standardized. For each sample ***x***, the log of each feature value *x*_*i*_ was taken and standardized by subtracting the median log feature value $\widetilde{{x_{i} }}~$ and dividing by the scaled interquartile range $\sigma _{i} = IQR/1.35~$ of the log feature value.

$$z_{i} = \frac{{\log \left( {x_{i} } \right) - \widetilde{{x_{i} }}}}{{\sigma _{i} }}$$

To generate permutations for local model training, values *z*_*i*_ were drawn from a normal distribution with mean 0 and width 1 and then transformed into feature values for Good and Poor permutations through

$$x_{{iG/P}} = {\text{exp}}\left( {\sigma _{{iG/P}} z_{i} + {}\widetilde{{x_{{i,G/P}} }}} \right)$$

where $\sigma _{{iG/P}} = IQR_{{iG/P}} /1.35$, $IQR_{{iG/P}}$ is the interquartile range for feature *i* as estimated from the Good (*G*) and Poor (*P*) VS reference samples separately, and $\widetilde{{x_{{i,G/P}} }}$ is the median log feature value as estimated from the Good (*G*) and Poor (*P*) VS reference samples separately.

Correlations between features were ignored. In total, 1 × 10^6^ permutations were generated: 5 × 10^5^ permutations were generated using median and interquartile range values taken from the 13 Good reference samples and 5 × 10^5^ permutations were generated using the 13 Poor reference samples. Permutations were classified as Good or Poor and those whose classification did not match the distribution from which they were drawn (~ 10% of the total) were discarded. Weights for each remaining permutation were calculated using a Gaussian distance kernel:

$$w = \exp \left( { - d^{2} /\sigma ^{2} } \right)$$

where the distance between the sample and permutation *d* is the Euclidian distance calculated in standardized space and the width $\sigma$ is the mean distance $\overline{{d~}}$ over all usable permutations (for logistic regression) or half the mean distance (for SVM). The local interpretable models (weighted logistic regression or weighted SVM) were then trained. The *LIME* explanations were taken as the regression coefficients of the standardized features for the logistic regression and coefficients of the standardized logged features of the vector perpendicular to the decision plane of the SVM.

It was not possible to train more local models, defined by smaller $\sigma ,$ for all instances in the cohort, as we need to adequately sample instances with both classifications for the training set. It was possible to reduce $\sigma$ to create explanations for instances very close to the boundary. These results, and others obtained for different parameter choices, are contained in Additional file 1: Figs. S11-S13.

## Results

### Exact Shapley values

**Fig. 1:**
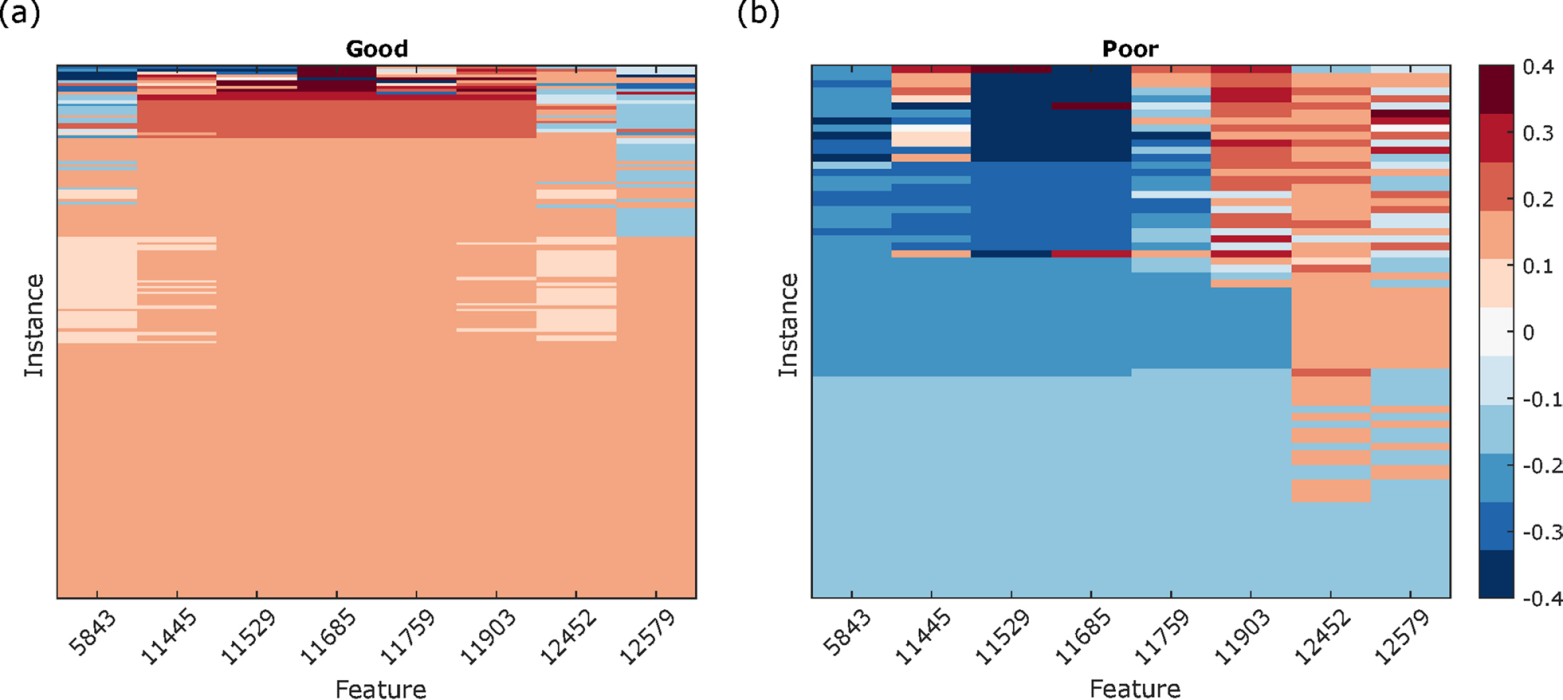
SV heatmaps for **a** Good and **b** Poor instances. Each row represents one instance and each column represents one feature. Rows (instances) are sorted by absolute value of the SV of the 11,685 feature, and this ordering is maintained throughout subsequent figures

Exact SVs for a single replicate (“instance”) classification result, generated for pretreatment samples for a cohort of 256 patients with NSCLC (189 Good, 67 Poor), were calculated according to the standard SV definition (Eq. 1). The Poor classification was taken to correspond to a numerical result of − 1 and the Good classification to a numerical result of + 1. The SVs for each of the eight mass spectral features (centered at 5843 Da, 11,445 Da, 11,529 Da, 11,759 Da, 11,903 Da, 12,452 Da, and 12,579 Da) are shown in the heatmaps of Fig. 1, separately for Good and Poor instances.

As expected from the concept of sample-specific feature importance, features contribute differently to the classification of different instances. While all instances in each respective heatmap (Fig. 1a or Fig. 1b) have the same classification, the VS 7NN algorithm does not arrive at these results using the features in the same way for all instances. However, some structure is apparent. Considering general patterns across instances, both Poor and Good heatmaps show that there is a substantial number of instances, more in the Goods (39%) than in the Poors (19%), where the SVs for all features are equal (with a magnitude of 1/8). We denote these as “uniform” Good/Poor instances. In terms of patterns across features, the features 11,529 and 11,686 have the largest SVs in general (more pronounced in the Poor instances (Fig. 1b)). The SV for the 11,685 feature is equal to the SV for the 11,529 feature for 246/256 instances, indicating equal importance of these two attributes. For almost all instances (255/256), the SVs for these two features have magnitudes of at least 1/8. We denote the ten instances where the SVs of these two features are not equal as “boundary” Good/Poor instances.

**Fig. 2:**
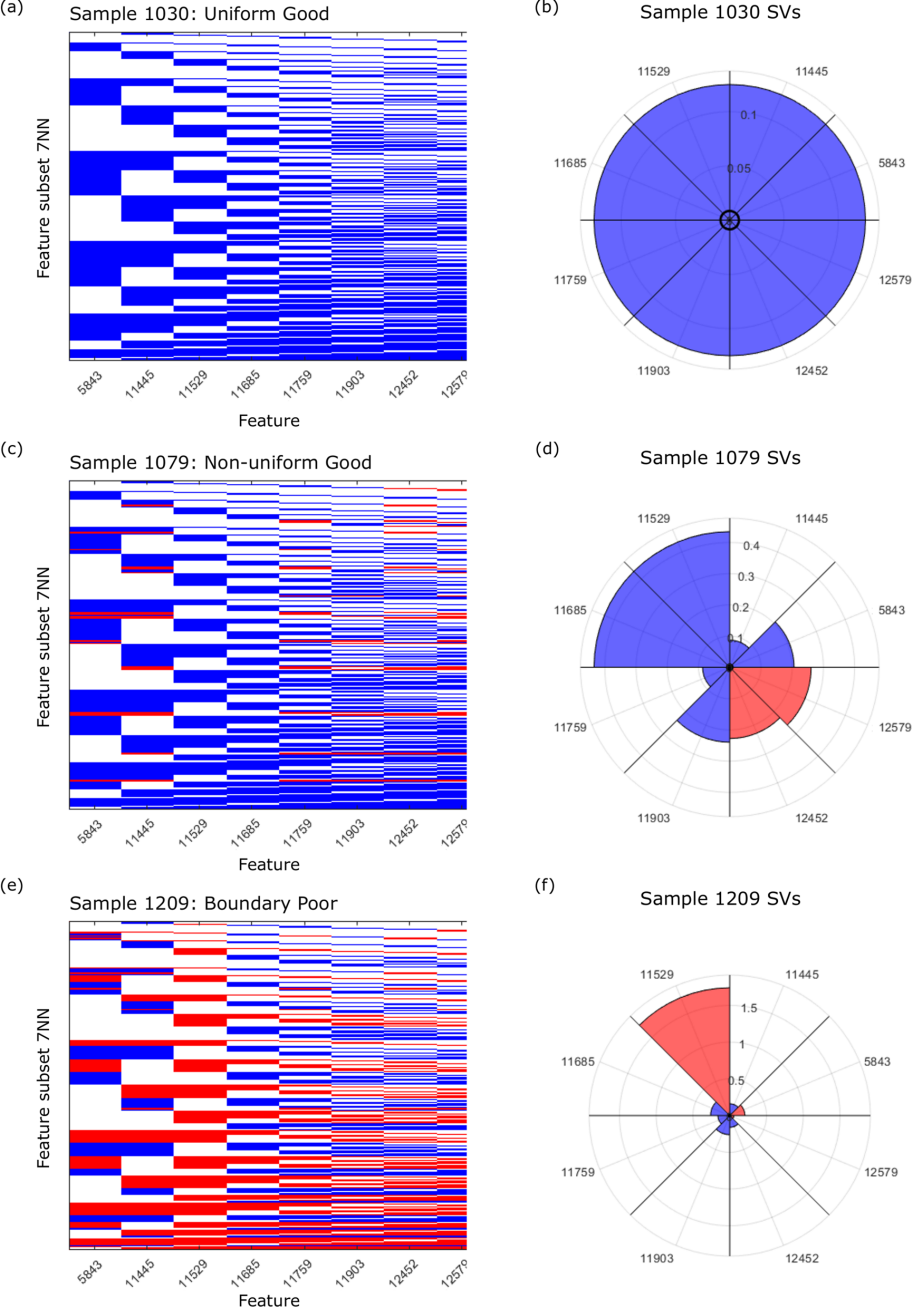
Schematic of the classifications of all 2^8^ (= 256) feature combination 7NN classifiers. We show **a** a uniform instance, **c** a nonuniform instance, **e** a boundary instance. Each heatmap row represents one feature subset 7NN classifier (increasing in subset size from top to bottom). Each column represents one of the eight features. Feature subsets not including the column feature are indicated in white; the 7NN classification result for feature subsets including the column feature are shown as Good (blue) or Poor (red). Corresponding radar plots (**b**, **d**, **f**) show the SVs for each instance. Magnitude is represented by radial extent and sign by color (positive as blue and negative as red)

We first discuss the interpretation of the SVs for the uniform instances. The uniform Good instances have all eight SVs equal to 1/8 and the uniform Poor instances have all eight SVs equal to − 1/8 (instances at the bottom of the heatmaps of Fig. 1). The equal SVs for these instances indicate equal importance of each of the eight features in the classification algorithm. The equal importance of all features is also seen in the example of Fig. 2a, in which the 7NN classification resulting from all possible subsets of features is shown for a uniform Good instance. Each column represents a feature, and each row represents a feature subset. Within each row, features not included in the subset are colored white, and those that are included are colored according to the classification that results from that row’s feature subset (blue for Good, red for Poor). For uniform instances (Fig. 2a), all feature subsets, except the null set (where the classification is uninformative = 0) produce the same classification as the full set of features, all features play equivalent roles and, via the SV axioms and Eq. 1, must have SVs of equal magnitudes of 1/8 (see e.g., Fig. 2b).

**Fig. 3:**
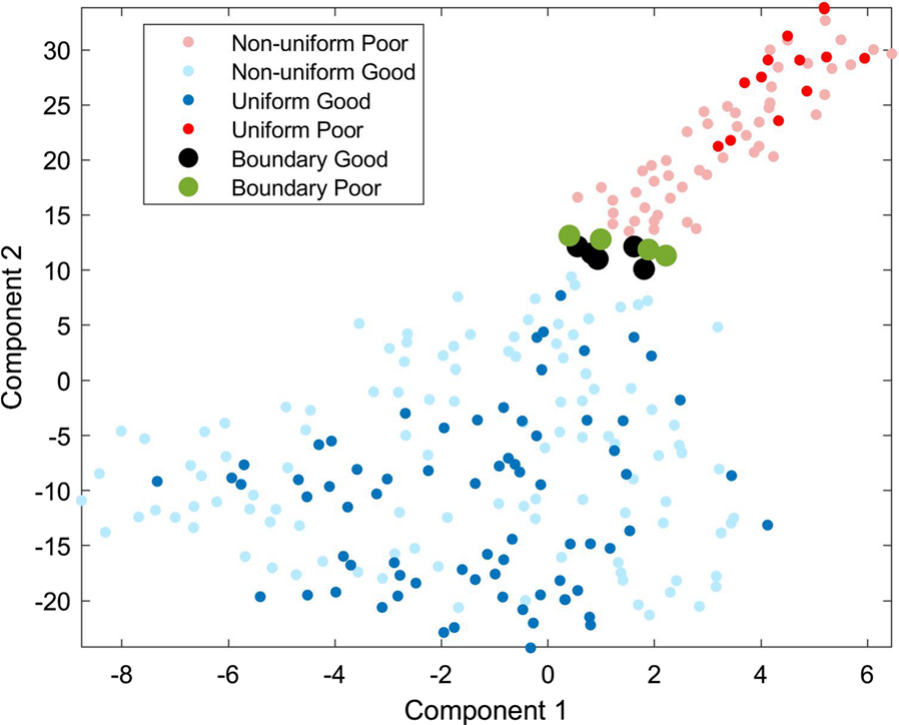
t-SNE plot of the eight VS features for all 256 instances. Uniform instances (|SVs|= 1/8) are shown in red (Poor) and dark blue (Good). Boundary instances are shown as large black (Good) and green (Poor) symbols. Other (non-uniform) instances are shown in pink (Poor) and light blue (Good)

Further understanding can be obtained from considering the position of the different instance types in feature space with respect to the decision boundary. Figure 3 shows a t-distributed stochastic neighbor embedding (t-SNE) plot [28] of the VS feature values for the 256 instances indicating uniform Good, uniform Poor, boundary Good, boundary Poor, and the remaining non-uniform Good and Poor instances. The Good and Poor instances separate into distinct regions. The uniform instances, with SVs of magnitude 1/8, lie in the bulk of the Good region and toward the far reaches of the Poor region.

The ten boundary instances, for which the SVs differ for the 11,529 and 11,685 features, lie on the boundary between Good and Poor classification regions of feature space in Fig. 3. For boundary instances, the 11,529 and 11,685 SVs can be of opposite sign, indicating that one feature influences classification towards a Good result and the other towards a Poor result. Differences in sign and large magnitudes are also observed for SVs of other features for boundary instances (see Figs. 1, 2 and Additional file 1: Fig. S8 for corresponding plots for additional instances). As the test decision boundary is approached, the SVs are sensitive to the interplay of the precise feature values, and small changes in feature value can lead to large changes in SV. This is not unexpected, given the strong nonlinearity of k-nearest neighbor (kNN) classification and the discontinuous nature of classification change at kNN decision boundaries [29].

The SVs for individual instances can also be represented by radar plots, which visually indicate the relative importance of features for the classification of an instance (Fig. 2b, d, f). For example, Fig. 2d illustrates that, even away from the decision boundary, features can act antagonistically in non-uniform instances: While the classification Good is dominated by the 11,529 and 11,686 features, features 12,579 and 12,452 would tend to classify this Good instance as Poor.

**Fig. 4:**
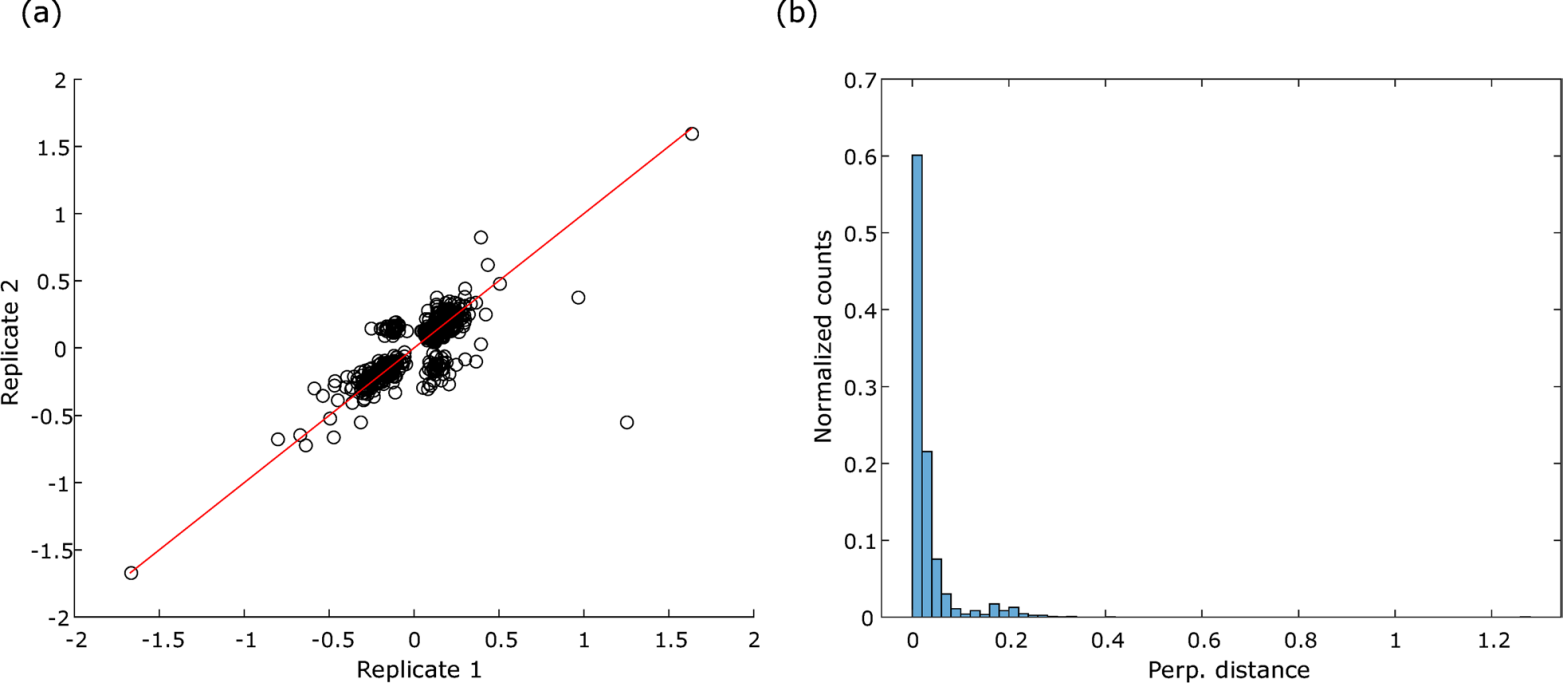
SV reproducibility between technical replicate 1 and 2 for 250 samples with concordant replicate classifications. **a** Replicate 1 SVs versus replicate 2 SVs for each feature and each sample. **b** Histogram of normalized counts (counts/#features x # samples) of perpendicular distance from each point in **a** to the y = x line

We assessed the reproducibility of SVs across samples with concordant replicate classifications (250/256 samples). SVs are compared between the first two replicates in Fig. 4 (other comparisons between the 3 replicates can be found in Additional file 1: Fig. S9). For most samples, SV reproducibility is very good, indicating a reliable and consistent attribution of feature importance for classification between technical replicates. A few samples, however, show larger variation in the SVs between replicates. These samples lie close to the test decision boundary.

### Exact Shapley interaction indices and Harsanyi dividends

**Fig. 5:**
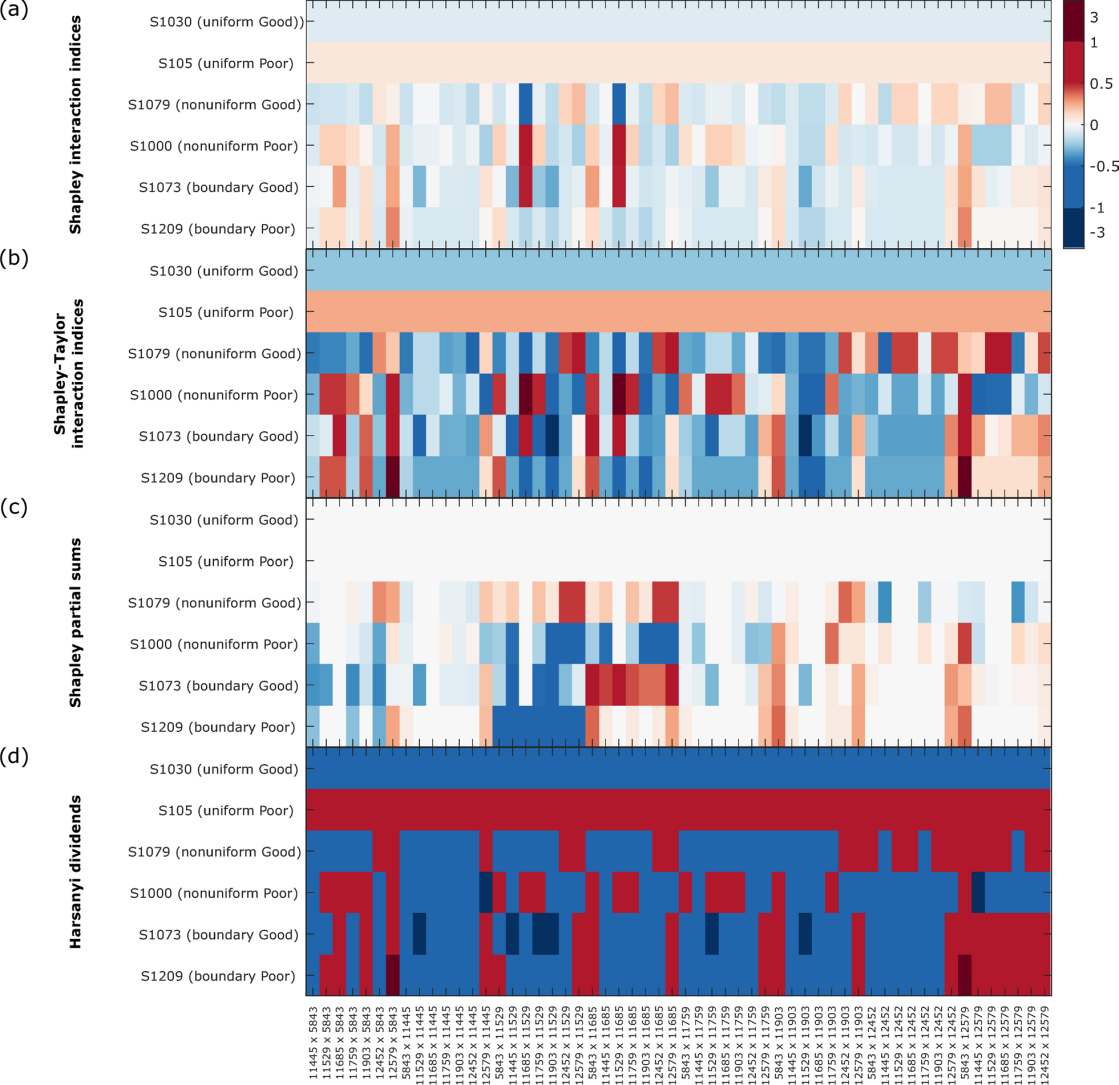
Heatmaps of interactions for representative instances. **a** Shapley interaction indices (*SIIs*), **b** Shapley-Taylor interaction indices (*STIIs*), **c** Shapley partial sums (*SPSs*), **d** Harsanyi dividends (*HDs*)

To assess the importance of pairs of features to the classification from the VS algorithm for each instance, we evaluated three previously proposed quantities: *SIIs* [14], *STIIs* [16], and *HDs* [7]. (Note that while *SIIs* and *STIIs* evaluate the contribution of features *i* and *j* in the context of coalitions of other features, *HDs* only consider features *i* and *j* in isolation.) The results are shown in the heatmap of Fig. 5 for all pairs of distinct features, $i,j~\left( {i \ne j} \right)$ for six instances: a uniform Good, a non-uniform Good and a boundary Good instance and corresponding examples of Poor instances.

For the uniform instances, the *SIIs*, *STIIs*, and *HDs* are − 1/14, − 1/4, and − 1 for Good instances and + 1/14, + 1/4, and + 1, for Poor instances, as can be seen from Eq. 2–Eq. 4 when all feature subset 7NN classifiers, apart from the empty subset, produce the same classification. Although the uniformity of the classifications is reflected by equal results for all pairs of features, the signs and magnitudes of the results do not reflect very intuitively the notion that the 7NN classifiers for feature subset $\left\{ i \right\}~ \cup S$, feature subset $\left\{ j \right\}~ \cup S$ and feature subset $\left\{ {i,j} \right\}~ \cup S$ all yield the same classification for all feature subsets *S* not including *i* or *j*. Non-uniform samples generally display less-intuitive interaction terms. The limited interpretability of the interaction terms is, at least in part, due to the categorical nature of the classification. In the case of this binary classification, each single feature subset classifier produces one of the two binary results. Consider the coalition of two features, each of which individually produces the same classification result as the full algorithm. The coalition of the two features can also only produce either that same result or the opposite result, i.e., it cannot produce a ‘better’ result than either of the single feature results. This leads to the interaction indices and Harsanyi dividends having opposite sign to the standard SV for the uniform instances.

*STIIs* provide a way of assessing main effect terms, in addition to the interaction between the two features. The main effect *STIIs* are + 1 or − 1, the classification for the individual feature 7NN classifier (Eq. 5). Main effect-like terms can also be constructed using the *SII* approach (Eq. 3), and these are ± 5/8 for Good and Poor uniform instances, respectively, for which the interpretation is again not very intuitive.

To assess the impact to classification of adding feature *i* to feature subsets including feature *j*, we calculated *SPS*_*ij*_. For uniform instances, the *SPS* is 0 for all $i,j~\left( {i \ne j} \right)$, corresponding to no change in classification from the inclusion of feature *i* in addition to feature *j* for all $i,j~\left( {i \ne j} \right).$ It also follows from Eq. 6 that $SPS_{{ij}} = 0~\left( {i \ne j} \right)$ for non-uniform instances when all 7NN subset classifiers containing feature *j* yield the same classification (e.g., 11,529 and 11,685 for sample 1079 and 11,529 for sample 1209 in Fig. 2).

### SHAP

We calculated *SHAP*-based approximations to SVs using kernel *SHAP*, the multivariate Gaussian approximation and the Gaussian copula approximation. For molecular data in general, and for the case of VS features in particular, we expect the multivariate feature distribution to be non-Gaussian with complex correlational structure. While we do not expect any real-world multivariate feature distribution to match the distributions which allow approximations of the conditional distribution needed for the *SHAP* approach, it is of interest to see how the results of these methods of increasing complexity compare with exact SVs.

**Fig. 6:**
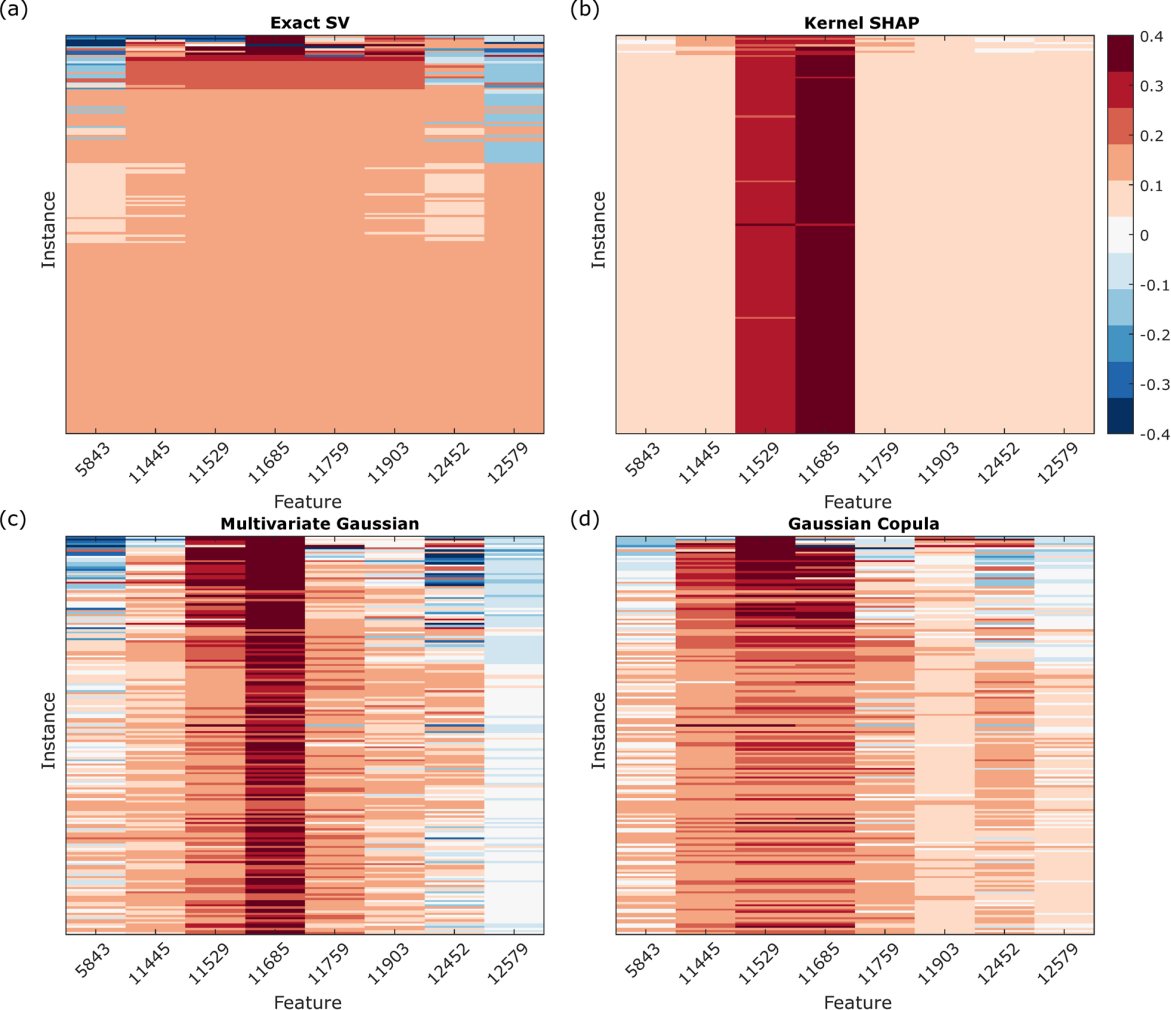
Heatmaps of exact SVs and *SHAP* values for the 189 Good instances. **a** Exact SVs, **b** kernel *SHAP* values, **c** multivariate Gaussian *SHAP* values, and **d** Gaussian copula *SHAP* values

**Fig. 7:**
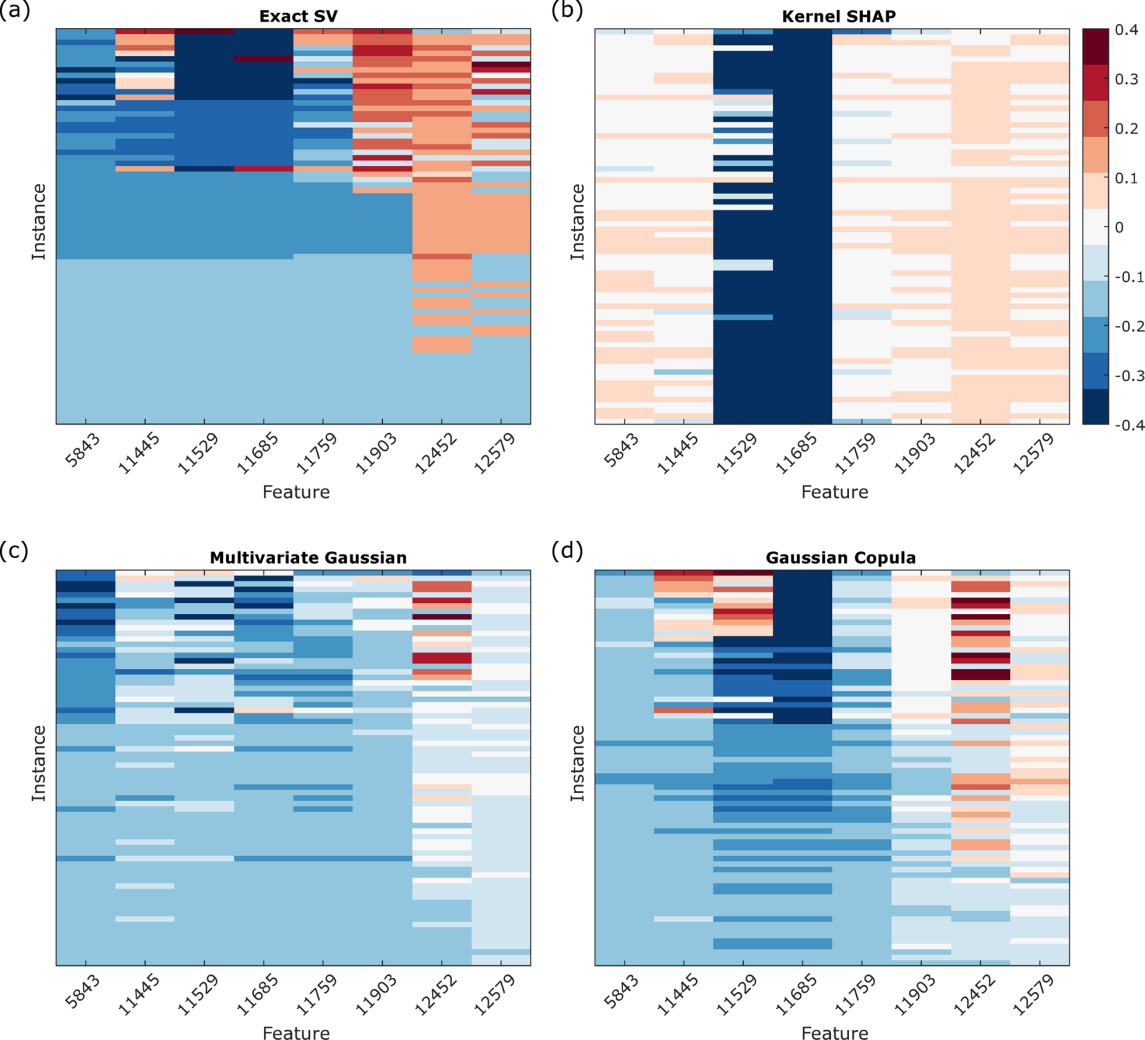
Heatmaps of exact SVs and *SHAP* values for the 67 Poor instances. **a** Exact SVs, **b** kernel *SHAP* values, **c** multivariate Gaussian *SHAP* values, and **d** Gaussian copula *SHAP* values

The SVs calculated using kernel *SHAP*, the multivariate Gaussian approximation and the Gaussian copula approximation are compared with the exact SVs for the 256-instance cohort in Figs. 6 and 7 for Good and Poor instances, respectively.

None of the *SHAP* approaches reproduce the exact SVs. Kernel *SHAP* identifies 11,529 and 11,685 as dominant features in classification to the almost entire exclusion of contributions of other features. This is likely due to the combination of these two features being largest in magnitude and the independent feature approximation meaning that the expected value is largely an average over sets of feature values never realized in the data obtained for real instances [26]. Kernel *SHAP* fails to recover the subset of instances with uniform exact SVs, and it produces *SHAP* values that are very similar for nearly all Good instances. Inclusion of some correlations between features via the multivariate Gaussian method weakens the extreme dominance assigned to the 11,529 and 11,685 features, especially in the Poor instances. The Gaussian copula approach, which allows for non-normal feature distributions, but maintains only pairwise correlations, further improves the *SHAP* values relative to the exact SVs. The mean square difference between the *SHAP* approximations and the exact SVs for the cohort, averaged over both instances and features, is 0.0455 for kernel SHAP, 0.0263 for the multivariate Gaussian approximation, and 0.0243 for Gaussian copula approach (Additional file 1: Fig. S10). We observed that for the multivariate Gaussian approach and the Gaussian copula approach, the mean square difference for the uniform instances between the results and the exact SVs were 0.0123 with standard error 0.0002 and 0.0044 with standard error 0.0003, where standard errors were evaluated over multiple subset samplings. Hence, even using the Gaussian copula approach, there remain substantial qualitative differences (fewer instances with uniform SVs, fewer instances with equal SVs for the 11,529 and 11,685 feature, lower SVs on average for 5843, 12,452 and 12,579 features) between the *SHAP* values and the exact SVs that could lead to different interpretations of the relative importance of the features for the classifications of many samples.

As it can be demonstrated that exact SVs are the only additive feature attributions that satisfy certain desirable axioms [6, 11] and the *SHAP* approximations differ from the exact SVs, at least one of the axioms must be violated by the *SHAP* approximations. Our results show violation of the symmetry, which requires that when two features contribute equally to all possible coalitions, i.e., when $f\left( {S \cup \left\{ i \right\}} \right) = f\left( {S \cup \left\{ j \right\}} \right)$ for every subset *S* that does not contain *i* or *j*, the SVs for the two features should be the same. From Fig. 2, we see that for the uniform sample (1079) and the non-uniform, non-boundary sample (1030), $f\left( {S \cup \left\{ {11529} \right\}} \right) = f\left( {S \cup \left\{ {11685} \right\}} \right)~$ for all *S* excluding features 11,529 and 11,685. However, none of the methods produces equal *SHAP* values for these samples, although the Gaussian copula *SHAP* values are close.

### LIME

**Fig. 8:**
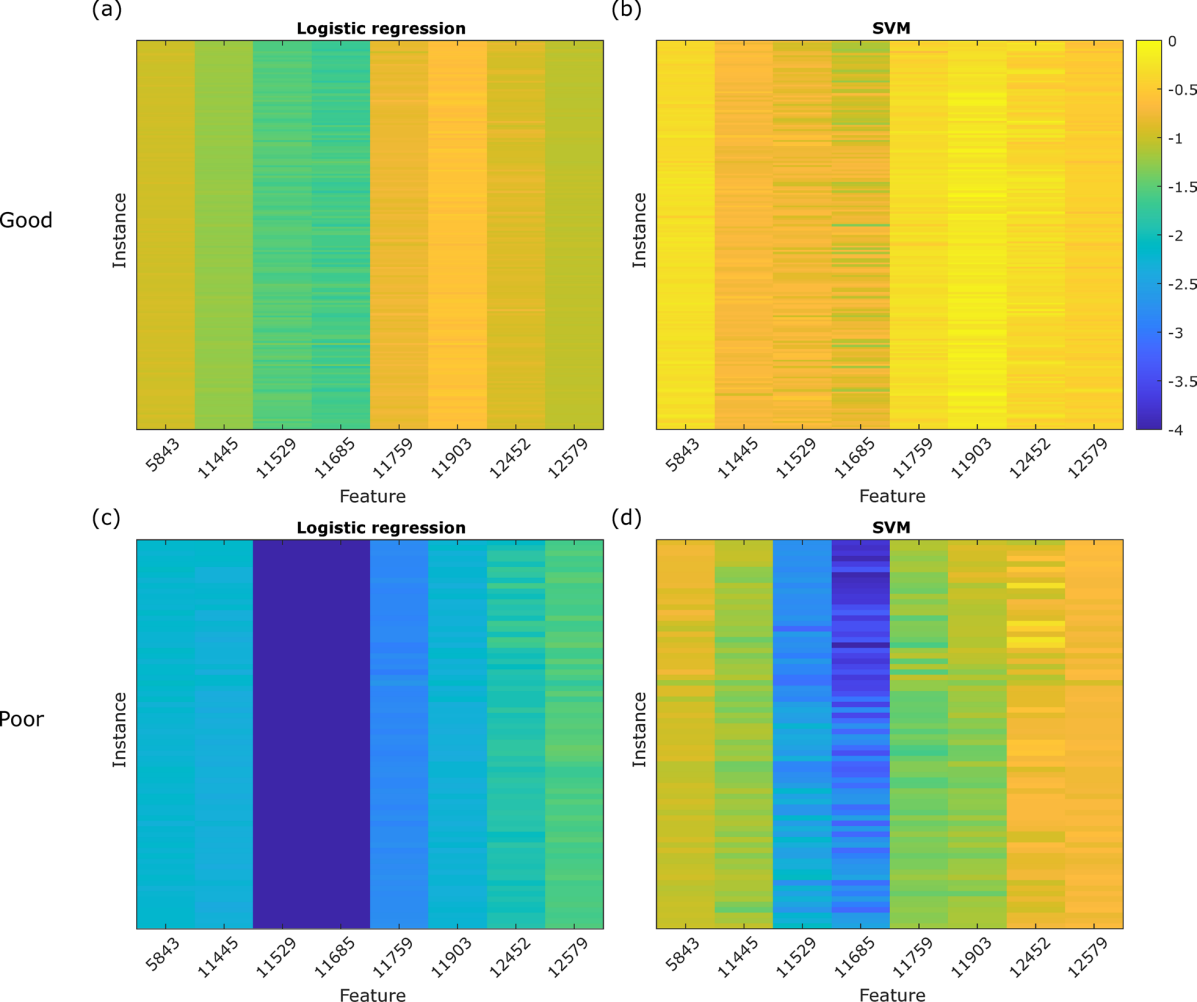
Heatmaps of *LIME* explanations. Logistic regression (**a** Good instances and **c** Poor instances) and an SVM (**b** Good instances and **d** Poor instances) are used as the local interpretable models

The *LIME* explanations obtained for the 256-instance cohort are shown in Fig. 8 for logistic regression and SVM as the local interpretable model. We see little variation in explanations across instances, reflecting the very loose definition of locality required to obtain explanations for all instances, as was discussed in the Methods section. Good instances show a very uniform relative feature importance across all eight features, while Poor instances are characterized by much stronger importance for the 11,529 and 11,685 features. In the boundary region, where we observed the boundary instances with their characteristic highly variable exact SVs, one can generate instances of different classification with local variation in features. However, it should be noted that here the VS algorithm is highly nonlinear, and a linear model is unlikely to provide an accurate local approximation.

While one does not expect a quantitative correspondence of *LIME* explanations with SVs, i.e., they are not a direct approximation, we do not observe even a good qualitative correspondence in feature importance assignment.

## Discussion

We have shown how the SV approach to model explainability can be applied to a molecular diagnostic test. The simple nearest neighbor algorithm used for the VS test allowed calculation of exact SVs for each of the eight VS features for a large cohort of samples obtained from a population of patients with NSCLC. The exact SVs provided an explanation of the relative importance of the eight features to the classification generated by the VS algorithm for each instance and showed good reproducibility between instances.

As was expected given the highly nonlinear nature of nearest neighbor classification, the relative importance of the features varied across the cohort. While the classification of a substantial proportion of instances (39% of Good instances and 19% of Poor instances) was controlled equally by all eight features, other instances had different patterns of feature importance, with instances close to the boundary between Good and Poor classified feature space regions showing the most variability and most extreme differences in SVs and hence most extreme differences in relative feature importance in determination of classification. Two features, 11,529 (related to C-reactive protein and isoforms of SAA) and 11,685 (C-terminal truncated SAA isoform) generally had more than average importance in determination of classification.

Being able to calculate exact SVs enabled us to compare these results with various approximations that are being increasingly used to provide information on feature importance for classification in cases where exact SV calculations are computationally prohibitive (see, for example, [30–33]). *SHAP* values were evaluated using kernel *SHAP* and two other extensions allowing incorporation of feature correlations excluded in kernel *SHAP*. None of these approaches produced results close to the exact SVs and they showed qualitative differences across the evaluation cohort.

While our specific example might be particularly challenging for these approximate methods, due to the highly nonlinear algorithm and the strong correlation between features, the qualitative and quantitative differences we observed from the exact SVs raise some concerns, given the ubiquity of correlations between features in clinical and -omics data and nonlinearities that facilitate knowledge extraction in modern ML. In addition, distributional-based approaches and methods that require assessment of correlations between features may be of limited utility in settings where training sets are small compared with the number of features used or are unrepresentative of patient populations. Both situations are not uncommon in clinical test development: practical constraints on clinical and observational study sizes often permit only relatively small test development cohorts and researchers sometimes compare extremes in outcomes rather than whole populations to elucidate features useful for classification and train ML algorithms. Hence, it might be wise to exercise caution in the interpretation of the results of *SHAP*-based approximations to SVs in the case of molecular tests using correlated features, especially if test algorithms are strongly nonlinear or training sets are small and allow only poor estimates of feature distributions and their correlational structure. Unfortunately, we are not aware of any methods that allow estimation of the deviations of *SHAP*-based feature importance attributions from exact SVs when the latter cannot be evaluated. Even if it is computationally expensive, it might be useful, if possible, to compute exact SVs for a few samples to compare with the approximate SVs; alternatively, results could be compared for a few samples between a desired approximation method and another with more relaxed distributional assumptions that requires more extensive compute time (e.g. multivariate Gaussian vs Gaussian copula), to provide at least a preliminary test of the reliability of the results of the simpler approximation method.

The *LIME* approach presented different issues in providing adequate explanations for feature importance for classification. The categorical nature of the VS classification, the well separated classification regions of feature space, and strong nonlinearity near the test decision boundary, all hampered definition of truly local interpretable models. Small training set size and lack of maintenance of feature correlations in local model construction would likely also be problematic when trying to use the *LIME* approach to explain other omics-based tests.

It is often of interest in a molecular diagnostic setting to examine the influence of pairs or subsets of features used simultaneously in a predictive algorithm. Hence, we investigated several quantities that have been proposed to assess feature interactions. *SIIs*, *STIIs*, and *HDs* could all be calculated exactly for the VS algorithm. However, the results were not very easy to interpret. We believe that this interpretational challenge arises from the binary nature of the VS classification; however, we anticipate that similar issues would arise for any categorical test and for classifiers with bounded outputs, for example when predicting risk or producing scores on a bounded scale. We proposed the idea of *SPSs* to examine the importance for classification of a feature *i* included in an algorithm in addition to a second feature *j* as an alternative metric for investigating the relative impact on classification of two features.

While SVs are useful to explain how patient data is converted to a test result by a specific test algorithm, it does not directly provide any information on the biological underpinnings of the test. Prediction is a task distinct from estimation and attribution, as discussed in detail by Efron [34], and good predictors can be created from molecular measurements or clinical attributes that do not directly represent the biological processes at play but are merely associated with them. Translational studies remain essential for exploring the biological mechanisms behind the performance of molecular tests. Nevertheless, SVs may inform such translational studies, because they may divide the groups of samples with identical test classifications into subgroups which use different patterns of features to arrive at the same classification label [14]. Even in the example of the VS algorithm, with only eight strongly correlated features, we observed uniform and non-uniform patterns of SVs, and one might expect to identify more SV-defined classification subgroups in tests that involve more molecular attributes. If one assumes that there are underlying biological mechanisms reflected in a classification, then a necessary corollary is that there may be different biological mechanisms corresponding to the different patterns of SVs. Hence, it could be of interest to study SV-defined classification subgroups using a translational approach in sample cohorts large enough to be able to reliably identify these subgroups and investigate their biological associations.

While SVs may inform feature importance considerations during an iterative test development process, we note that SVs can only reflect the feature importance in determining the classification for a specific classifier. Other classifier architectures may be more or less efficient in combining attributes, combine attributes in different ways, and may lead to different patterns of SVs. There are also challenges in using SVs for feature selection or feature optimization for classifier development when features are correlated, as discussed in detail by Kumar et al. [26]. Hence, the SVs for a particular model cannot, in general, reliably predict whether the performance of the model will get better or worse if certain features are excluded or included; however, it will be the case that when $\psi _{i} = 0$ for all instances, feature *i* does not play any role in classification and therefore could be excluded from the model. There are also additional factors that should be considered in test design and development, and features may be included in a test based not only on their relative importance for generating a prediction, but also on their ability to improve the reproducibility or robustness of the test. There remain important aspects of test interpretability that cannot be addressed directly by SVs, including causality [26], which could be important for patients, physicians and regulators [35].

We have illustrated some limitations of approximations to SVs and hence shown the utility of being able to calculate exact SVs. It has already been observed that certain ML architectures facilitate SV calculations, e.g., tree*SHAP* [14]. The additive axioms satisfied by SVs facilitate SV calculations for tests based on ensemble averages, and ML methods based on regularized combinations of small coalitions of features also present the possibilities of exact SV calculations for tests which include large numbers of features [36–39]. Systematic studies of the convergence of sampling-based approximations to exact SV calculations for models with these architectures are underway.

## Conclusions

Exact SVs can be used to determine the relative importance of features to the classification generated by a multivariate model for a specific sample or patient. In the same way that physicians diagnose some diseases using different weightings of clinical factors for individual patients, the patterns of SVs may vary between samples receiving the same classification from a molecular diagnostic test. Patient subgroups defined by these SV patterns may be of interest for translational research, because they may reflect different biological mechanisms giving rise to the same classification result. *SHAP* approximations may differ both qualitatively and quantitatively from exact SVs when correlations between features are important, and so, in general, caution is advised when applying *SHAP* approximation methods to explain the results of molecular diagnostic tests.

This study discusses the interpretation of SVs, interaction SVs, and the differences between exact SVs and various commonly used approximations to SVs using the example of one molecular diagnostic test with specific ML architecture and training set feature distribution. Although the general principles of utility and interpretation of exact SVs and interaction SVs will not depend on the details of the test to be explained, it would clearly be of interest to investigate calculation of SVs and differences between exact SVs, *SHAP*-based SV approximations and *LIME* in a large scale, systematic study that would investigate different ML architectures and span attribute distributions with various correlational structures, via use of synthetic datasets and multiple types of real-world molecular data. Future research could also focus on how best to present SV information to physicians and patients and how to use SV analysis to inform translational studies designed to address the mechanisms of action of molecular diagnostic tests and fundamental biology of the disease indications in which the tests are employed.

### Availability and requirements

The datasets generated and/or analyzed during the current study are available from the corresponding author on reasonable request.

## Supplementary Information

**Additional file 1**. Supplementary information on method implementation and additional results

## Data Availability

The datasets generated and/or analyzed during the current study are available from the corresponding author on reasonable request.

## Abbreviations

7NN: 7-Nearest neighbor
AI: Artificial Intelligence
HD: Harsanyi dividends
kNN: K-nearest neighbor
LIME: Local interpretable model-agnostic explanation
ML: Machine learning
NSCLC: Non-small cell lung cancer
PIT: Probability integral transform
SAA: Serum amyloid A
SHAP: Shapley additive explanations
SII: Shapley interaction indices
SPS: Shapley partial sum
STII: Shapley-Taylor interaction indices
SV: Shapley value
SVM: Support vector machine
t-SNE: T-Distributed stochastic neighbor embedding
VS: VeriStrat
