# Supplementary material for: Explaining multivariate molecular diagnostic tests via Shapley values
Source: BMC Med Inform Decis Mak. 2021 Jul 8;21:211. doi: 10.1186/s12911-021-01569-9 (PMC8265031; doi:10.1186/s12911-021-01569-9)

**Additional coalition diagrams and radar plots**

**Figure 8: Schematic of the results of all 2^8^ feature combination 7NN classifiers for (a) a uniform poor instance, (c) a nonuniform poor instance, (e) a boundary good instance. Each row represents one feature subset 7NN classifier (increasing in subset size from top to bottom). Each column represents one of the eight features. Feature subsets not including the column feature are indicated in white; the 7NN classification result for feature subsets including the column feature are shown as Good (blue) or Poor (red). Corresponding radar plots ((b),(d),(f)) show the SVs for each instance. Magnitude is represented by radial extent and sign by color (positive as blue and negative as red).**

**
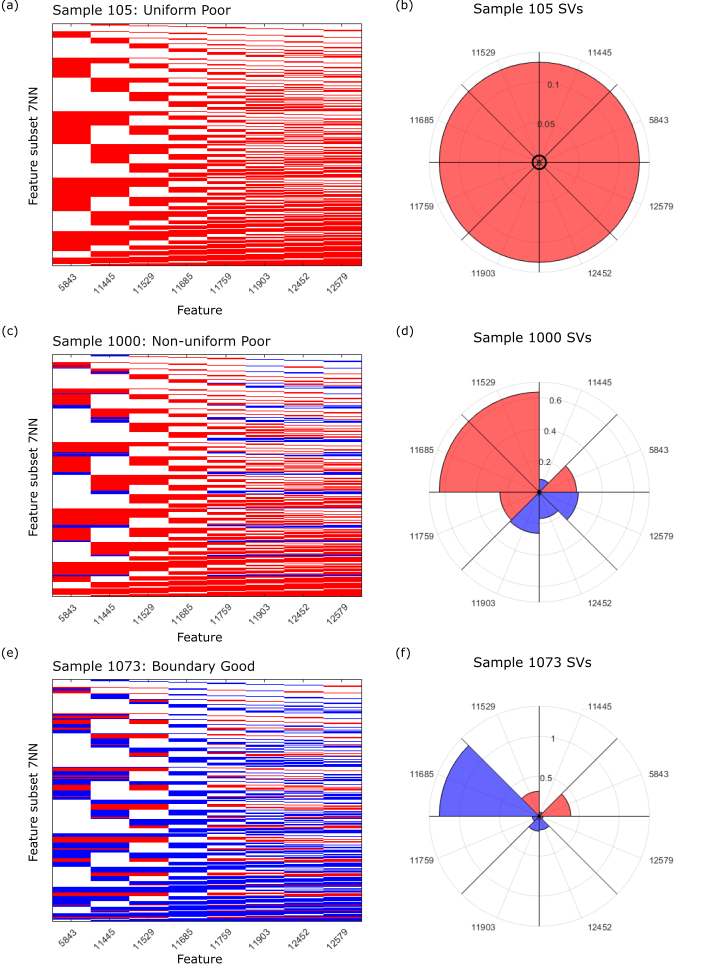
**

**Additional Results on Reproducibility**

**Figure 9: Reproducibility of the SVs across technical replicates for all 256 samples. (a) Replicate 3 SVs plotted against replicate 1 SVs. (b) Histogram of log counts of perpendicular distance from y=x line in (a). (c) Replicate 3 SVs plotted against replicate 2 SVs. (d) Histogram of log counts of perpendicular distance from y=x line in (c).**

**
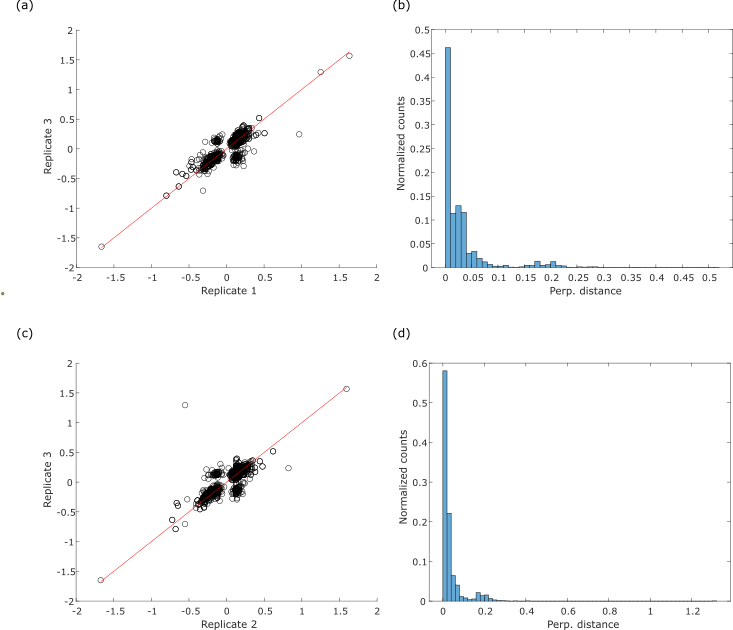
**

**Additional SHAP information**

**Figure 10: Mean square difference or error (MSE) between exact SVs and kernel, multivariate Gaussian, and Gaussian copula SHAP values**

**
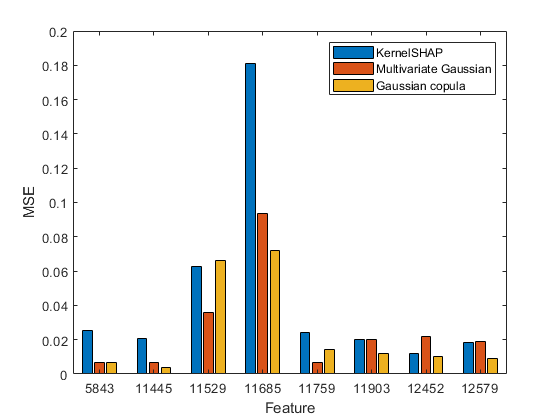
**

**Additional LIME methods**

*Logistic regression, all reference samples used, features assumed to be uncorrelated*

Permutations were generated from standardized feature values as described in the main text, but all $1\times{10}^{6}$ permutations were generated using the median and scaled interquartile range (IQR/1.35) taken from the entire reference set of 26 samples. Weights were calculated using a Gaussian distance kernel and Euclidean distance from the sample using standardized feature values. The kernel width was the mean distance over all permutations.

*Logistic regression, all reference samples used, permutations drawn from multivariate Gaussian distribution*

$1\times{10}^{6}$ permutations were generated from a multivariate Gaussian distribution as described in the main text and in Aas et al, using the median and variance of the entire reference set of 26 samples. Weights were calculated using a Gaussian distance kernel and Euclidean distance from the sample using standardized feature values. The kernel width was the mean distance over all permutations.

*Logistic regression, all reference samples used, permutations drawn from Gaussian copula distribution*

$1\times{10}^{6}$ permutations were generated from a Gaussian copula distribution as described in the main text and in Aas et al, using the median and variance of the entire reference set of 26 samples. Weights were calculated using a Gaussian distance kernel and Euclidean distance from the sample using standardized feature values. The kernel width was the mean distance over all permutations.

**Figure 11: Heatmaps of** $\boldsymbol{LIME}$ **explanations for Good instances with the entire reference set used to generate permutations (a) Features assumed to be uncorrelated, (b) Permutations drawn from a multivariate Gaussian distribution, (c) Permutations drawn from a Gaussian copula distribution.**


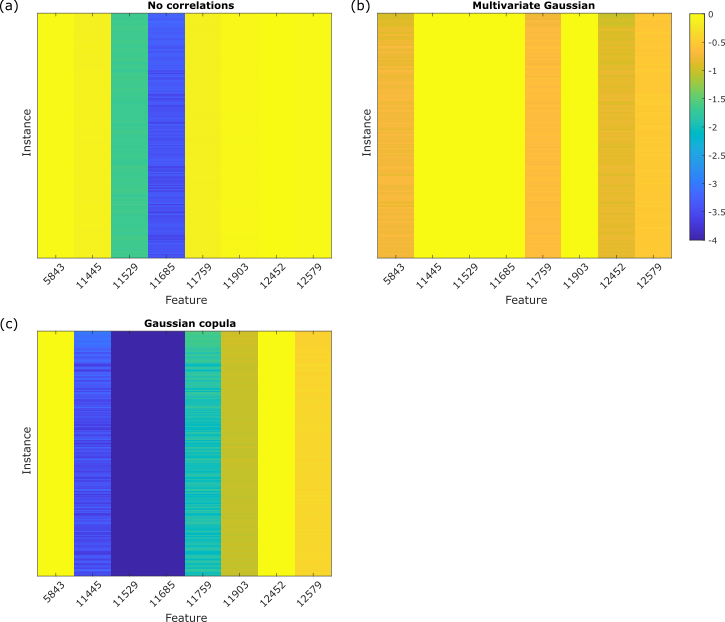


**Figure 12: Heatmaps of** $\boldsymbol{LIME}$ **explanations for Poor instances with the entire reference set used to generate permutations (a) Features assumed to be uncorrelated, (b) Permutations drawn from a multivariate Gaussian distribution, (c) Permutations drawn from a Gaussian copula distribution.**


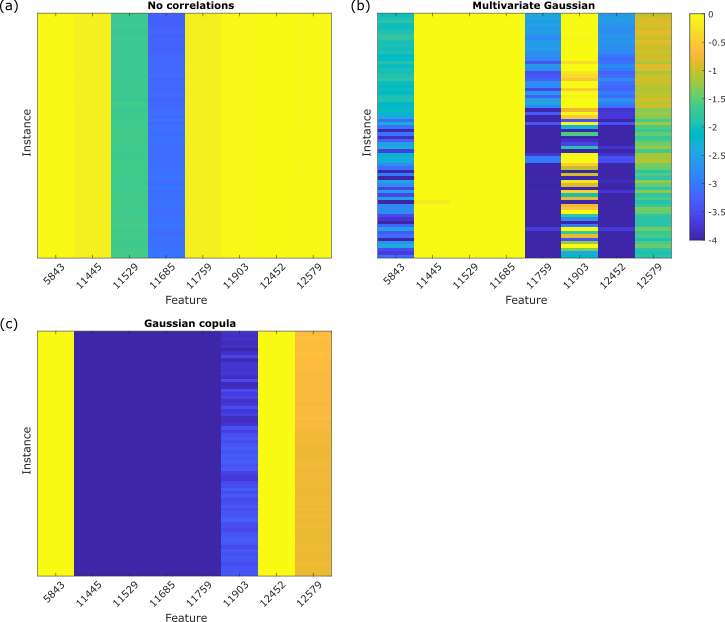


**Figure 13: Heatmaps of** $\boldsymbol{LIME}$ **explanations for boundary samples with varying width of the Gaussian distance kernel** $\boldsymbol{\sigma}$**. (a) Width equal to the mean distance between sample and permutation. (a) Width equal to half the mean distance between sample and permutation. (a) Width equal to one-fifth the mean distance between sample and permutation.**


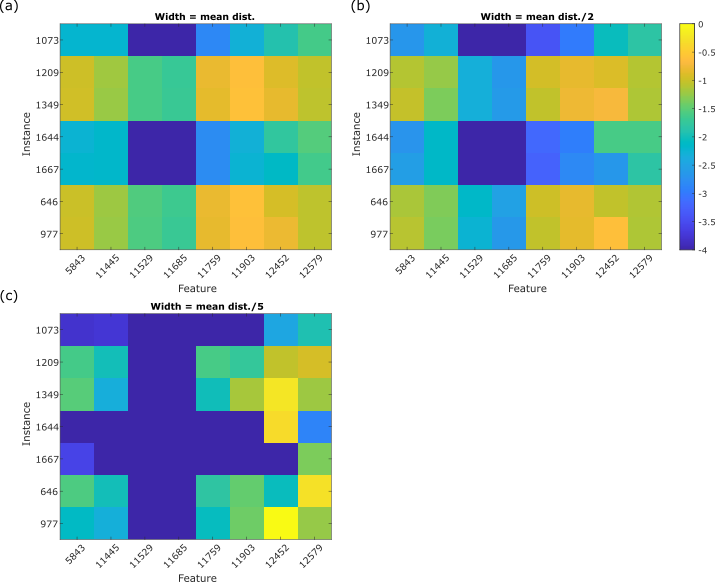

Supplement: Supplementary file 1 — Additional file 1. Supplementary information on method implementation and additional results [file 12911_2021_1569_MOESM1_ESM.docx]
